# Biology, systematics, and clinical manifestations of Zygomycota infections

**DOI:** 10.1007/s10096-014-2076-0

**Published:** 2014-03-11

**Authors:** A. Muszewska, J. Pawłowska, P. Krzyściak

**Affiliations:** 1 Institute of Biochemistry and Biophysics, Polish Academy of Sciences, Pawiskiego 5a, 02-106 Warsaw, Poland; 2 Department of Plant Systematics and Geography, University of Warsaw, Al. Ujazdowskie 4, 00-478 Warsaw, Poland; 3 Department of Mycology, Chair of Microbiology, Jagiellonian University Medical College, 18 Czysta Street, 31-121 Krakow, Poland

## Abstract

Fungi cause opportunistic, nosocomial, and community-acquired infections. Among fungal infections (mycoses) zygomycoses are exceptionally severe, with a mortality rate exceeding 50 %. Immunocompromised hosts, transplant recipients, and diabetic patients with uncontrolled keto-acidosis and high iron serum levels are at risk. Zygomycota are capable of infecting hosts immune to other filamentous fungi. The infection often follows a progressive pattern, with angioinvasion and metastases. Moreover, current antifungal therapy frequently has an unfavorable outcome. Zygomycota are resistant to some of the routinely used antifungals, among them azoles (except posaconazole) and echinocandins. The typical treatment consists of surgical debridement of the infected tissues accompanied by amphotericin B administration. The latter has strong nephrotoxic side effects, which make it unsuitable for prophylaxis. Delayed administration of amphotericin and excision of mycelium-containing tissues worsens survival prognoses. More than 30 species of Zygomycota are involved in human infections, among them Mucorales is the most abundant. Prognosis and treatment suggestions differ for each species, which makes fast and reliable diagnosis essential. Serum sample PCR-based identification often gives false-negative results; culture-based identification is time-consuming and not always feasible. With the dawn of Zygomycota sequencing projects significant advancement is expected, as in the case of treatment of Ascomycota infections.

**Electronic supplementary material:**

The online version of this article (doi:10.1007/s10096-014-2076-0) contains supplementary material, which is available to authorized users.

## Introduction

Zygomycota is an artificial grouping of distantly related basal fungi. This term is broadly used for fungi belonging to Mucoromycotina, Entomophthoromycotina, Mortierellomycotina, Zoopagomycotina, and Kickxellomycotina. The common name zygomycosis indicates infections caused by organisms that diverged more than 800 MYA [1], preceding land colonization by plants. Consequently, it should be broadly recognized that zygomycoses have a variable prognosis depending on the causal agent and patient’s condition. Mycology is undergoing a significant shift toward molecular-based taxonomy, aiming at a more consistent classification and nomenclature. A robust classification reflecting biological similarities is of great significance for medical purposes. The biochemical and physiological variability among fungal taxa lead to measurable effects, among them antifungal resistance [2, 3]. Fast and accurate species (or at least genus) recognition is essential for a successful treatment in the case of less common fungi. Yet, in most laboratories such in-depth classification is not routinely performed, because these genera cannot be easily differentiated, for example, histopathologically.

The broadly used and recognized term Zygomycota should be abandoned in the future, in favor of proper taxonomic names. Since the clinical manifestations and development of infections with Mucorales and Entomophthorales are significantly different, the name “zygomycosis” should also be abandoned in favor of entomophthoramycosis (entomophthoromycosis), mortierellomycosis and mucoralomycosis (mucormycosis). However, here, when describing the common features of many taxa, we will apply the term Zygomycota. Emerging Zygomycota infections are becoming a serious threat at transplantation and on other wards with immunocompromised patients. This phenomenon become more pronounced after the introduction of serum-based detection, diagnostic imaging, and modern antifungals (voriconazole) that enabled efficient treatment of aspergillosis [4]. Mucorales are the third most common agent of systemic mycoses after invasive aspergillosis and candidiasis in hematology patients [5]. This constantly growing group of susceptible patients, together with improvements in the management of invasive aspergillosis and candidiasis result in increased interest in zygomycoses. In contrast to aspergillosis, most cases of zygomycosis are fatal, despite the administration of antifungal therapy [6–8]. The number of at-risk patients is constantly growing, with the ubiquity of diabetes and immunosuppressive treatment. Furthermore, Zygomycota are able to colonize hosts not susceptible to other opportunistic fungal infections, among them diabetic patients and intravenous drug users [8]. In particular, representatives of the genus Basidiobolus [9] and of the family Saksenaeaceae [10, 11] have been reported to infect healthy hosts.

Here, we revise current knowledge on nomenclature, pathogenesis, and diagnostics of zygomycosis (mainly mucormycosis).

## Biology

Mucoromycotina representatives are ubiquitous organisms present all over the world in the soil, infecting plants and other fungi [12]. Importantly, the same species are found in clinical samples and used in food fermentation. Entomophthoromycotina groups more than 250 entomopathogenic, soil, and litter-associated species [13]. Mortierellomycotina [14] harbor more than 100 taxa of saprotrophic soil organisms.

The knowledge on pathogenic Zygomycota ecology is very limited and often anecdotal. Understanding the lifestyle of pathogenic species is essential to understanding the epidemiology and to formulating recommendations for at-risk patients. In medical mycology species from the order Mucorales are the most common Zygomycota representatives.

Mucorales reproduce both sexually, by zygospores formed after the fusion of hyphae of different mating types, and asexually through sporangiospores produced within sporangia. However, in most species zygospores production is rarer and the conditions necessary for their formation and germination remain unknown. In turn, asexual spores are produced in massive quantities and they can differ in character [15]. Mucorales produce not only dry sporangiospores dispersed by the air, but also wet sporangiospores, less prone to aerosolization. Spore size and hydrophilic/hydrophobic character impacts the dispersal of fungi. Zygomycetes that infect humans are relatively hydrophilic, which limits everyday exposure to their spores, even if they are ubiquitous in spoiled food and their pathogenic strains do not differ from environmental samples [16].

Even the most ubiquitous among Zygomycota (*Rhizopus* and *Mucor*) are less common in air samples than the benign, plant-associated *Alternaria* and *Cladosporium* [17, 18] involved in human allergies. The prevalence of Mucorales spores in dust and spoiled food compared with air samples is consistent with the onset of infections after earthquakes and massive inhalation of spores. Human pathogenic Ascomycota are broadly encountered in air samples, whereas Zygomycota are less abundant in air samples [19–21]. In liquid culture *Mucor circinelloides* produces yeast cells; however, the role of yeast forms in infection is unknown [22]. Furthermore, *M. circinelloides* produces sporangiospores of different sizes depending on the mating type variant [23]. The bigger sporangiospores are able to lyse macrophages, which makes them more virulent to a mammalian host.

Until recently, it was not clear whether the dimorphic switch between aerobic hyphal growth and multi-budded yeast growth under anaerobic/high CO_2_ conditions is related to virulence in Mucorales. Lee and colleagues [24] showed that a calcineurin inhibitor tacrolimus (FK506) prevents hyphal growth of *Mucor* spp. Furthermore, the cAMP-dependent protein kinase A (PKA) is involved in the dimorphic transition as in other dimorphic fungi (*Candida* species, *Cryptococcus neoformans*, *Cryptococcus gattii*, and *Aspergillus fumigatus*). The yeast-locked mutants were less virulent than wild-type strains, suggesting that hyphae are more virulent or the dimorphic switch is important for the pathogenicity of this fungus. Finally, the authors showed that calcineurin participates in hyphal polarity development and spore size dimorphism. Calcineurin is a promising drug target against many fungal pathogens with dimorphic growth (Table 1).

**Table 1:** Summary of differentiating features among filamentous fungi

Character	Ascomycota (e.g., *Aspergillus*)	Zygomycota (e.g., *Mucor*)
Spores	Hydrophobic spores (conidia), ascospores	Dry and wet sporangiospores, zygospores
Hyphae	Narrow (2–3 μm), septate, branching in different angles	Broad (6–16 μm), coenocytic, orthogonal ramifications
Cell wall composition	More glucan	More chitin
	Galactomannan present	Galactomannan absent

Mucoralean hyphae are often heavy and have a bigger diameter than in Ascomycota. Hyphae diameter and size determine the efficacy of phagocytosis by macrophages. They differ in cell wall composition as well, and they have less glucan and more chitin compared with Ascomycota. This change in biochemistry determines antifungal susceptibility/resistance, because most antifungal drugs target either the cell wall machinery or components. They are galactomannan-negative; thus, serum tests based on this feature can differentiate between Ascomycota and Zygomycota, but only to confirm the presence of Ascomycota and they provide no information about the presence/absence of Zygomycota. Zygomycota are resistant to most of the newer antifungals, such as voriconazole and echinocandins, which are very efficient against *Candida* and *Aspergillus* mycoses. The three orders of Zygomycota infecting humans have different susceptibility/resistance patterns.

In vitro studies revealed the potential for biofilm formation for *R. oryzae*, *L. corymbifera*, and *R. pusillus*, but not for *A. elegans* [25]. The biofilm matrix is formed with glucosamine as the dominant dry component (Table 2).

**Table 2:** Summary of differentiating features of entomophthoromycosis and mucormycosis

Feature	Mucormycosis	Entomophthoromycosis
Progression of the infection	Rapidly invasive	Local
Immune reaction	Acute	Chronic
Host immunity	Compromised	Competent
Localization	Systemic	Cutaneous, soft tissue
Lipase	−	+
Keratin degradation	−	+
Thermotolerance	+	−

*Rhizopus oryzae* is claimed to possess extraordinary genome plasticity owing to a whole genome duplication, which resulted in an elevated number of genes, including those involved in host–pathogen interactions [6, 26]. Currently, more Zygomycota genomes have been sequenced, including *Lichtheimia hyalospora*, *Mucor circinelloides*, *Mortierella elongata*, *Rhizopus microsporus*, and *Conidiobolus coronatus*, which will enable comparative studies and accelerated screening of drug targets. Comparative analyses will facilitate the identification of conserved components of the fungal cell machinery. Based on the sole gene count and genome size comparisons, it is clear that *Rhizopus oryzae* is an exception regarding the number of genes in the whole collection of currently sequenced Zygomycetes, with the average gene number of 11 thousand genes. Table 3 lists the estimated genome sizes and predicted gene numbers for Zygomycetes present in the Joint Genome Institute (JGI) sequencing center database.

**Table 3:** Currently sequenced Zygomycota genomes stored by the Joint Genome Institute (JGI)

Organism and project name	Genome size	Number of genes
*Coemansia reversa* NRRL 1564 v1.0	21,838,014	7,347
*Umbelopsis ramanniana* AG # v1.0	23,077,072	9,931
*Conidiobolus coronatus* NRRL28638 v1.0	39,903,661	10,635
*Rhizopus microsporus* var. *microsporus* v1.0	25,972,395	10,905
*Mucor circinelloides* CBS277.49 v2.0	36,587,022	11,719
*Lichtheimia hyalospora* v1.0	33,282,407	12,062
*Gonapodya prolifera* v1.0	48,794,828	13,902
*Mortierella elongata* v1.0	49,959,475	14,964
*Phycomyces blakesleeanus* NRRL1555 v2.0	53,939,167	16,528
*Rhizopus oryzae* 99-880 from Broad	46,087,117	17,467

## Systematics

Fungal systematics have been recently reorganized [27], which resulted in temporary chaos in the nomenclature. A revised taxonomic classification has been introduced for several of the human and animal fungal pathogens that are also part of the previous Zygomycota phylum [12–14]. These changes in nomenclature should better reflect biological differences among taxa. A reliable source of valid nomenclature are the Index Fungorum and MycoBank. Zygomycota themselves do not currently constitute a valid taxon; instead, more subphyla and orders are proposed.

Only 3 of the 13 orders of known Zygomycetes, namely the Mucorales, the Mortierellales, and the Entomophthorales, harbor animal and human pathogens [28]. However, *Mortierella wolfii* is a cattle pathogen, the documentation of human infections is scarce and no cases have been described since 2000. Consequently, we will focus only on Mucorales and Entomophthorales infections, called mucormycosis and entomophthoromycosis respectively). Supplementary Table S1 lists the Mucoromycotina, Mortierellomycotina, and Entomophoromycotina that are involved in human infections with synonymic names and examples of case reports. The mucoromycotina group contains most of the human pathogenic Zygomycota.

Some taxa have been recently revised, deemed to be species complex and consequently divided into more taxa. Among them, *Apophysomyces* spp. now account for four taxa with differences in spore shape and ITS sequence levels [29]. Owing to the introduction of separate taxa in 2010, most reports in the literature refer to *A. elegans*. However, future work should refer to the newest nomenclature. A similar pattern involves *Saksenaea vasiformis*, which is now considered a species complex and in consequence was split into more taxa characterized by spore shapes [30]. Among the most characteristic pathogenic Mucorales *Rhizomucor variabilis* was moved to the Mucor genus based on molecular characteristic and renamed *Mucor irregularis* [31]. Other taxa suspected to be species complex were confirmed as true species. *Rhizopus microsporus* was revised as a single species and its varieties might be just environmental variants. Importantly, foodborne, environmental and clinical strains are indistinguishable [32]. This observation supports the opportunistic character of mucormycoses

## Host—fungus interaction

Key factors involved in fungal infections seem to be shared among distantly related fungi pathogenic to different hosts [24, 33].

The respiratory tract is the main entrance for fungi, among them Zygomycetes. Inhaled aerosolized spores are removed by the movements of ciliated epithelial cells. If some manage to overcome this barrier the alveolar macrophages phagocytose and destroy most spores. Healthy gastrointestinal tract and skin are good barriers to Zygomycetes. It is not surprising that most skin infections are a consequence of direct inoculation due to severe trauma. Gastrointestinal infections are most common in neonates, who have not yet developed proper immune mechanisms.

The description of host pathogen interactions at a molecular level shows differences in the mechanisms of aspergillosis and mucormycosis, including host immune response and susceptibility to macrophage-induced hyphal damage [34]. Furthermore, biological features of the causing agent determine the location and progress of the infection, leading to a more localized infection in entomophthoromycosis and a progressive and potentially disseminated infection in mucormycosis. Entomophthorales specialized in cutin degradation exert keratinolytic enzymes; as a consequence entomophthoromycoses are usually superficial and gastrointestinal infections. In contrast to Entomophthorales, human-infecting Mucorales are usually thermo-tolerant, which makes them able to invade internal body parts and form a chronic infection.

The interplay between pathogens and hosts varies among fungal agents and the host condition. However, some general trends can be drawn. Most of the mechanisms are known from animal data; the latter should be considered with caution as the inflammatory reaction is different in rodents and apes. The role of the immune system in preventing and eliminating fungal infections involves an inflammatory reaction. Inflammation triggered by Zygomycota is usually less visible than in aspergillosis. Ascomycota are recognized by both TRL2 and TRL4 receptors, whereas Zygomycota are recognized solely by TLR2 receptors [34]. There is another major difference compared with aspergillosis regarding the involvement of T lymphocytes. This is because innate immunity is indispensable in fighting a Zygomycota infection and the acquired immunity involvement is not that pronounced. However, Mucorales-specific T cells and NK cells can be found both in patients and in healthy individuals. These findings pave the way for potential diagnostic tests and adoptive immunotherapy [35].

One of the most characteristic features of zygomycoses is their occurrence in diabetic patients. Some aspects of the interplay between the fungus and diabetic patients has been revealed at a molecular level. During the infection *R. oryzae* binds to GRP78 receptors [36, 37]. The expression of GRP78 receptor coding genes is affected by acidosis and by iron and glucose levels, which are observed in diabetic patients (high blood sugar levels and available iron). In such a scenario *R. oryzae* become more resistant to neutrophils, which are susceptible to low pH conditions. One taxon may produce hyphae and spores of variable size. Phagocyte-induced damage is related to target mass; bigger hyphae are harder to phagocytose. Schmidt and colleagues [38] demonstrated that both unstimulated and IL-2-prestimulated human NK cells damage *Rhizopus oryzae* hyphae, but do not affect resting conidia. They concluded that the damage of the fungus is mediated, at least in part, by perforin. *R. oryzae* hyphae decrease the secretion of immunoregulatory molecules by NK cells, such as IFN-γ and RANTES (regulated on activation, normal T-cell expressed and secreted), indicating an immunosuppressive effect of the fungus.

A study on the activity of human polymorphonuclear leukocytes (PMNLs) against *R. oryzae*, *R. microsporus*, and *L. corymbifera* revealed that interferon (IFN)-gamma and granulocyte-macrophage colony-stimulating factor (GM-CSF) augment the hyphal damage of all three zygomycetes. Additionally, this effect was more pronounced against *L. corymbifera* than against both *Rhizopus* species [39].

Genus-specific patterns could be drawn from pulled data from multiple infections. It is currently known that Cunninghammella is more aggressive than *Lichtheimia* in diabetes patients [34]. It induces low TNFα levels and is resistant to hyphae damage by macrophages. In rare cases it can invade an immunocompetent host [40], which suggests some of the specific properties of *Cunninghammella* compared with other Mucorales. *Cunninghammella* infections have higher mortality rates than other, more common infections [8].

## Epidemiology

### Risk factors

There are several risk factors associated with opportunistic fungal infections, among them zygomycoses. Diabetic ketoacidosis [8, 41], treatment with corticosteroids [42, 43], deferoxamine in patients on dialysis [44], and immunosuppressive drugs [36] enhance susceptibility to developing zygomycosis. Neutropenia [36, 45], malnutrition [46], cytomegalovirus infection [47], and extended wounds/burns [48, 49] are also predisposing conditions associated with zygomycosis.

According to the main reviews by Roilides et al. [34] and Ibrahim et al. [36] neutropenia, diabetes with blood hyperglycemia and low pH, Fe overload, corticosteroid therapy, and direct inoculation as a result of trauma are the main risk factors. Neutropenic patients are at increased risk, whereas patients with AIDS are not. This is because T lymphocytes are not key players in fighting against Zygomycota infection. The phagocytes provide antifungal activity via oxidative metabolites and defensins [36]. Immunosuppression leading to neutropenia encountered in transplant recipients renders them susceptible to zygomycosis. Corticosteroid therapy also influences neutrophils and their activity against fungi.

It has been found that [8] diabetic patients are exceptionally susceptible to sinusitis infections. Diabetic patients have an altered innate immunity with reduced chemotaxis of PMNLs, impaired transmission through the vascular endothelium, and reduced superoxide production [50, 51].

With regard to Fe overload, iron is indispensable for vital life processes and as a such is a limiting factor. Pathogens developed many traits to acquire iron from the host and Zygomycetes are no an exception. Deferoxamine therapy is suspected to facilitate Zygomycota infection by making iron available.

Healthy skin provides an efficient barrier against Mucorales; thus, skin trauma provides a risk of developing mucormycosis [36]. This condition is favorable for fast-growing opportunistic organisms. Contact with soil, food, and water containing fungi can be detrimental to either burned or severely wounded patients (Table 4).

**Table 4:** Infection prevalence and its main features [8, 41, 52, 53]

Underlying disease/condition	Percent of patients with zygomycosis	The most common form of infection	Mechanism and predisposing factors
Hematological malignancy	11–17	Pulmonary; disseminated	Neutropenia, voriconazole prophylaxis
HSCT recipients	1, 2–5	Rhinocerebral; pulmonary	Steroid prophylaxis for GvsH disease, neutropenia, voriconazole prophylaxis
Diabetes mellitus	17–36 (74)	Rhinocerebral, sinus	Acidosis—increasing iron level acidosis and hyperglycemia—has a negative impact on neutrophil chemotaxins and phagocytic activity
Solid organ transplantation	(0.6) 7–13, 23	Pulmonary, liver, and other transplant organ	Transplant contamination
Deferoxamine therapy	1–6	Disseminated, pulmonary	Increasing iron level
Burns, trauma in accident	13–20	Cutaneous, even 40 % of infections are disseminated	Disruption of skin barrier (spore inoculation)
Malnourished patients	3	Gastrointestinal	Digestion of the fungi
(Premature) neonates	3–21	Gastrointestinal	

### Sources and routes of infection

Mucorales are ubiquitous in the environment. They may be present in rotten organic debris (foods, fruits, vegetables, seeds and nuts, plants). Sporangiospores are easily aerosolized and dispersed in the air, which may be the source of infection by inhalation of spores. However, the occurrence of sporangiospores in indoor and outdoor air is less than that of conidia of aspergilli and other molds.

There are three main routes of infection for Zygomycota. The most common is via the respiratory system, i.e., the sinuses and lungs. Wards where there are patients with neutropenia and other risk factors for invasive fungal infection should undergo air-conditioning monitoring, and plants, which are potential sources of fungal growth and sporulation, are not permitted there.

Published data describing the levels of zygomycete sporangiospores in outdoor and indoor air in relation to seasonal variation are scarce. It was suggested on the basis of two articles from the Near East and analyses of the seasonal presence of sporangiospores in outdoor air that mucormycosis may occur seasonally at the end of the summer and in the fall, when the weather is warm and humid [21].

The second route of infection is via direct inoculation of fungal propagules into the human body. It can be iatrogenic or commonly acquired. Nosocomial infection can be due to contaminated medical devices, e.g., ostomy pouching systems, adhesive bandage, wooden tongue depressors, subcutaneous insulin infusion pump, peritoneal dialysis, intravascular devices, or contamination during medical procedures, e.g., dental extraction and local anesthesia, intramuscular injection of corticosteroids, vitamins and anticoagulants, nasal packing, contamination of grafts, and during transplantation. Commonly acquired inoculation of zygomycota fungi may occur as a result of traffic accidents, burns when the contaminated soil, water, plant debris among others, comes into contact with injured skin and tissues. Infection can also occur through insect bites. This route of infections is dominant in healthy patients. The less common type of Zygomycota infection is via the oral route [51].

Among nosocomial sources of infection there are reported cases of contaminated biomedical devices and building constructions. In a review of 169 cases by Rammaert and colleagues [51] a variety of sources of infections are listed, among them grafts, ostomy bags, adhesive bandages, water, wooden tongue depressors, dental extraction and local anesthesia, blood glucose self-monitoring equipment, subcutaneous insulin infusion pump, peritoneal dialysis, intravascular devices, intramuscular injection of corticosteroids and vitamins, and nasal packing. Neonatalogical, hematological, transplantation and burn units should monitor their conditions to ensure patients’ well-being.

Special focus should be directed toward donor-derived filamentous fungal infections in transplant recipients [54], which have a fatal outcome [51]. Environmental sources of infection are dominant in healthy patients with a challenged skin barrier—after trauma (car accident, burns), among them soil/decaying organic matter [55] and water [56] should be considered. Even plant pots in hospitals can be a source of infection for immunosuppressed patients.

### Prevalence

In the general population, zygomycosis is a very rare disease. Orphanet (the Orpha number, i.e., the Orphanet identifier for zygomycosis is 73263) classified it as a rare infectious disease; however, its prevalence is not known. There are several estimations of the incidence of zygomycosis/mucormycosis, showing some discrepancies. A more accurate assessment of zygomycosis prevalence is currently not feasible owing to incomplete identification and documentation. However, some best practice approaches have been introduced, like the Working Group on Zygomycetes by the International Society for Human and Animal Mycology (ISHAM). Rees and colleagues [57] estimated the incidence of mucormycosis to be 1.7 cases per million people per year, analyzing a population-based study in the USA performed in the 1990s. A Spanish, multicenter, population-based study, based on 6 diagnosed cases of zygomycosis in Spain in 2005 [58], showed that the incidence of zygomycosis was 0.43 out of 1,000,000 inhabitants per year and 0.62 out of 100,000 hospital admissions. That gives 430–1,700 cases per million per year worldwide. However, Brown [59] estimated that more than 10,000 life-threatening cases of mucormycosis occur worldwide per year, with 30–90 % mortality rate within infected persons.

Postmortem prevalence evaluation shows that mucormycosis is 10- to 50-fold less frequent than both candidiasis and aspergillosis, with a frequency of 1–5 cases per 10,000 autopsies [60–62]. Zygomycosis represents 8.3–13 % of all fungal infections encountered at autopsy in high-risk patients [52].

Males are more likely to be affected by zygomycosis than women (58–68 % of cases [8, 63–65]). This fact can be explained by the incidence of hematological malignancies (HM) (and most cancers) being generally lower in women than in men. The smaller number of cancers in women is a well-documented phenomenon and could be in part the result of a lower exposure to environmental and occupational risk factors in women than in men [66, 67]. Omoti et al. [68] reports that HM affects male (56 %) and adult (>15 years; 83 %) patients more often. However, Skiada et al. found that there is no predilection as regards sex in cutaneous zygomycosis, which affected generally healthy subjects [69].

In 2011, a large prospective multinational European study based on 230 patients with zygomycosis showed that the most common predisposing conditions were hematological malignancies (44 %), trauma (17 %), DM (17 %), and hematopoietic SCT (9 %) [65].

#### Hematological malignancies

Patients with hematological malignancies (HM) either with or without hematopoietic stem cell transplantation (HSCT) constitute the most commonly infected group. They account for up to 44 % of all cases of zygomycosis [45, 52].

The retrospective cohort study was conducted in the hematology wards of either tertiary care centers or university hospitals located throughout Italy, between 1999 and 2003. Five hundred and thirty-eight proven or probable invasive fungal infections (IFI) in 11,802 patients with newly diagnosed hematology malignancies (AML, ALL, CML, CLL, NHL, HD, and MM) were documented. The overall incidence of IFI was 4.6 %. Zygomycetes in this study occur in 1.2 out of 1,000 (0.12 %) HM patients (most frequently among AML and ALL patients—0.3 %) with 64.3 % mortality rate (attributable mortality rate 0.08 %). In this study zygomycetes were the fourth most common agents of IFI following *Aspergillus*, *Candida*, and *Fusarium*) [7].

In another study conducted in Southern Italy over 18 months, in 589 patients (475 adults and 114 pediatrics) with newly diagnosed HM (AML, NHL, ALL, CLL, MM), 27 episodes of IFIs were documented [70]. The overall incidence was 4.6 %. Of the 27 documented IFIs, 16 were caused by yeasts (13 in adult patients and 3 in pediatric patients) and 11 by filamentous fungi (all in adult patients). A total of 11 mold infections (40.7 %, 10 aspergillosis and 1 zygomycoses) were reported only in adult patients (incidence 2.3 %). Occurrence of zygomycosis was slightly higher than observed in the study by Pagano et al. [7]—1.6 out of 1,000 vs 1.2 out of 1,000 HM patients.

Neofytos and colleagues [71] performed a multicenter, prospective, observational study to assess the epidemiological characters and outcomes of invasive fungal infection (IFI) in hematopoietic stem cell transplant (HSCT) recipients in North America. In this study were included 234 adult HSCT recipients with a total of 250 cases of IFI. The most frequent was invasive aspergillosis (59.2 %) followed by invasive candidiasis (24.8 %) and zygomycosis (7.2 %), and other molds (6.8 %) [72]. In 178 autopsies of HSCT recipients (45.1 %) infectious etiologies were found to contribute to the cause of death. Fungal infections were found in 93 cases (23.5 %). Among fungal infections caused by cultivable species the most common was *Aspergillus* spp. (44.6 %) followed by *Candida* (33.9 %), Zygomycota (7.1 %), and *Pneumocystis* (7.1 %).

These data suggest that zygomycetes infection occurs in 1.7 % of patients with HSCT recipients (17 cases of zygomycosis per 1,000 HSCT recipients).

#### Diabetes

Another risk factor is diabetes mellitus. Zygomycosis occurs mainly in patients with uncontrolled diabetes, especially those with ketoacidosis. In 2012 the WHO estimated the number of diabetic patients to 220 million (current estimates from the WHO Fact sheet no. 312, updated March 2013: 347 million people worldwide have diabetes [73]). Zygomycoses are associated with DM in 17–36 % of cases. If we take into account estimates that worldwide there are 10,000 patients with zygomycosis, which gives us 0.9-1.8 cases of zygomycosis per 100,000 diabetic patients (approximately 0.0009–0.0018 %).

The problem of zygomycosis in diabetic patients is associated with poverty and malnutrition, for example, in India where uncontrolled DM was the most commonly found predisposing factor in 74 % (*n* = 131) of the 178 cases of zygomycosis identified between 2000 and 2004 [74]. In contrast to France, where DM was associated with only 9 % of 531 cases of zygomycosis reported between 1997 and 2006 [63].

#### Solid organ transplantation

Zygomycoses have been reported from different organs such as kidney, liver, heart and lung transplantations. Zygomycosis was generally considered to be a rare complication in solid organ transplant recipients [75]. Incidence of zygomycosis in solid organ transplant recipients is estimated to range from 0.4 to 16 % depending on the procedure and geographical area [63]. The most recent multicenter prospective study conducted in the USA found that out of 16,457 transplant patients 12 had Mucorales infection (0.07 %); among organ recipients in particular, Mucorales infection occurred as follows: lung 2 out of 1,179 (0.17 %), liver 7 out of 4,361 (0.16 %), kidney 3 out of 8,494 (0.03 %); in the pancreas (1,174), heart (1,159), and small bowel (69) there were no zygomycosis infections [5].

Although zygomycoses are relatively rare they are characterized by a severe course and high mortality rate. Early diagnosis of the infection is still a challenge and the demand for better diagnosis is growing with the increasing number of reported cases. The latter is possibly caused by a species shift resulting from the common prophylaxis of fungal infection with azoles (fluconazole and voriconazole) in at-risk wards. Zygomycota are not susceptible to azoles, except for posaconazole.

### Clinical manifestations

There are five main clinical manifestations of Zygomycosis [52]: rhinocerebral, pulmonary, gastrointestinal, cutaneous, and disseminated. Spellberg and colleagues listed six groups of miscellaneous mucormycosis presentation in which they included brain involvement without sinus infection, endocarditis, pyelonephritis, bone infection, external otitis, etc. [53].

Among patients from the group at risk of zygomycosis, the most common manifestation is rhino-(orbital)-cerebral (33–49 %), followed by cutaneous (10–16 %), pulmonary (10–11 %), disseminated (6–12 %), and gastrointestinal (2–11 %). In immunocompetent/otherwise healthy patients the most representative clinical form was the cutaneous/subcutaneous form (42.5 % of patients), followed by the rhino-orbito-cerebral (38 %) and genitourinary (8.5 %), disseminated, and pulmonary infections [76].

#### Rhinocerebral mucormycosis

Rhinocerebral mucormycosis is the most common form present in both neutropenic hematological and diabetic patients. Initial symptoms are nonspecific, involving headaches, altered mental status, fever, and eye syndrome, lacrimation, irritation, or periorbital anesthesia. Unilateral vision disturbance and further changes involving ptosis, proptosis or loss of extraocular muscle function are signs of the progressing infection toward the retro-orbital region or the central nervous system (CNS). Necrotic black lesions on the hard palate, necrotic turbinates, and septum perforation should be carefully inspected. The radiographic image of the infection can be mimicked by bacterial and other fungal infections as well as tumors. Scheckenbach and colleagues [77] reported that initial CT scanning was not informative and most manifestations were not specific; only histopathology provided sufficient data to establish a reliable diagnosis. Proper management of the primary disease is essential for a favorable outcome.

#### Pulmonary mucormycosis

Pulmonary mucormycosis usually occurs in patients with profound neutropenia, hematological malignancies, those on corticosteroid therapy or those with diabetes. The symptoms are not specific and involve fever, chest pain, and cough. Neutropenia and coagulation dysfunctions make tissue collection inadvisable in some cases. Radiographic or CT scans showing multiple (>10) nodules or pleural effusions can be a sign of fungal infection. Although other mycoses, bacterial infections or malignant invasions should be considered.

#### Gastrointestinal mucormycosis

Gastrointestinal mucormycosis is a very rare form of infection. It is associated with severe malnutrition and premature birth. The infection is thought to be a consequence of the ingestion of fungi. The symptoms including fever, pain, vomiting, diarrhea, and constipation are nonspecific. The infection may lead to ischemic infarction and ulceration. The stomach, terminal ileum, and colon are the most common sites; however, any part of the gastrointestinal tract can be colonized. The diagnosis requires direct examination and biopsy, and is often only performed at autopsy.

#### Cutaneous (skin) mucormycosis or entomophthoromycosis

Cutaneous infection can be either primary or secondary. A primary infection develops in patients who have suffered burns or other trauma. The infection is caused by direct inoculation during an accident or can be nosocomial. The disease manifestation involves erythema, pus, abscess formation, tissue swelling, necrosis, and pain in the infected area. The skin appears red and indurated and the necrosis can be observed as black eschars. The tissue necrosis can progress to gangrenous cellulitis.

Cutaneous infection can be a secondary locus in patients with disseminated infection from the respiratory tract. Cutaneous mucormycosis has been reported in diabetic patients.

#### Disseminated mucormycosis

This form of mucormycosis is the hardest to control and constitutes the greatest threat to the patient. When two or more noncontiguous organs are involved an infection can be considered disseminated. These often include lung and CNS colonization, with the lung often being the primary infection site. The involvement of the lung and skin is a sign of a higher risk of CNS invasion as well. Since the disseminated form often involves CNS colonization, the disease can manifest as changes in the mental status. Other internal organs can be secondarily invaded during colonization, among others the spleen, liver, and even the heart, leading to pain in the infected organ.

#### Pediatric cases

Zaoutis and colleagues consider that pediatric cases should be treated as a separate category [78, 79]. There is limited information about zygomycoses in children. Infection in children is different: they tolerate more intensive treatment and GI tract infections are fatal, being the third most common manifestations of zycomycoses in neonates.

### Diagnosis of Zygomycota infections

Zygomycosis is still a challenge for diagnostics, especially considering that early diagnosis is the best drug in zygomycoses. The diagnosis relies on clinical findings, risk factor analysis, histopathology, and culture samples inspection. The clinical signs vary with the form and stage of the infection. In rhinocerebral zygmycosis, classic clinical features include facial swelling, with ocular involvement. Patients often report facial and eye pain, proptosis, and visual disturbances that are signs of the involvement orbital structures (muscles, nerves, and vessels). Black necrotic lesions can also occur, for example, on the hard palate. Pulmonary zygomycosis manifests with nonspecific symptoms such as fever, cough, and dyspnea; when fungi invade vessels hemoptysis may also occur. Radiographic findings can be vague and often overlap with other mold infections. The presentation of pulmonary zygomycosis often resembles that of an invasive aspergillosis in severely immunocompromised patients, for example, HSCT recipients. Comparison of the CT imaging features of pulmonary zygomycosis and invasive pulmonary aspergillosis has shown that the presence of multiple nodules (>10) and, to a lesser degree, the presence of pleural effusions, favored the presence of the former diseases. Additionally, micronodules seen on the initial CT scans were more commonly observed in patients with pulmonary zygomycosis [80]. Gastrointestinal zygomycosis may occur in patients with severe malnutrition. The clinical picture mimics intra-abdominal abscess. Unfortunately, the diagnosis is often made at autopsy. Differentiation with other fungal infection is very important because Zygomycota are resistant to voriconazole, a drug successfully used in the treatment of invasive aspergillosis.

The diagnosis can be performed on several levels:

1. Identification of an invasive mold infection
2. Distinguishing between Ascomycota and Zygomycota
3. Species/genus identification

### Diagnostic imaging

Diagnostic imaging is useful in the early diagnosis of rhinocerebral, non-enhanced paranasal sinuses or lung lesions. This method allows the preliminary recognition of probable Mucorales infection. Computed tomography (CT) has a higher sensitivity and allows an earlier diagnosis than the classical radiographic techniques. CT scans can reveal lesions caused by angioinvasive fungi not visible on conventional radiographs and before other clinical manifestations [81]. CT gives the opportunity to show mucosal thickening and early bone destruction. Lesions that could be revealed on lung CT scans are nodules, halo signs, reverse halo signs, cavities, wedge-shaped infiltrates, pleural effusions, and angioinvasion of molds. These need to be confirmed by biopsy and molecular or culture-based methods. The presence of pulmonary infiltrates in neutropenic patients is already a sign of angioinvasion, necrosis, thrombosis, hemorrhage, and edema. These can be the first observable signs of angioinvasion and systemic fungal infection. CT imaging of infected organs often shows limited inflammation around hyphae compared with *Aspergillus*. The observed lesions can change in the course of infection, but are not specific enough to reliably differentiate among causing agents like *Aspergillus*, *Scedosporium*, *Fusarium*, Mucorales, and mixed infections. The major disadvantage of using CT is the exposure of patients to a high dose of radiation. The radiation exposure associated with CT scans limits their routine or serial usage.

Another imaging technique used in diagnostics is MRI. This method is mandatory for assessing extension into the cavernous sinus and identifying cerebral involvement. Lesions that can be revealed by MRI are orbital and intradural expansion, cavernous sinus thrombosis, or thrombosis or aneurysm of the internal carotid artery.

Positron emission tomography (PET) has the highest sensitivity among diagnostic imaging methods. This technique can detect small infectious foci before the onset of the anatomical abnormalities assessed by conventional radiology tools. PET is not recommended for the evaluation of cerebral involvement and in diabetic patients control of glycemia is also required.

Preliminary diagnosis based on the above-described techniques needs to be confirmed by biopsy and molecular or culture-based methods.

### Direct examination and culture-based methods

Direct examination can provide only limited information on the causative agent, but in the absence of the quick diagnostic test, biopsy with histopathological assessment remains a crucial method in the diagnosis of zygomycosis. In tissue materials zygomycosis can be recognized by the presence of broad, nonseptate hyphae, invading tissue and causing necrosis, vascular invasion, and thrombosis. The hyphae of Zygomycota branch at a right angle, as opposed to other fungi such as *Aspergillus*, whose hyphae branch at a slight angle. However, they can be easily overlooked in tissue samples stained with nonfungal staining. Many infections are diagnosed definitively only at autopsy.

To diagnose mucormycosis different specimens are taken that are adequate to the location of the ongoing process of infection. For the rhinocerebral form the appropriate samples are as follows: sinus aspirates, tissue specimens from the affected area, and also scraping of the nasal mucosa. In the case of pulmonary infection deep samples of tissues and bronchoalveolar lavage (BAL) with flexible fiber optic bronchoscopy can be obtained. Sputum testing is less sensitive, especially in non-neutropenic patients. CT-guided biopsy can also be performed in the case of negative BAL findings [82].

The biopsy of the lesion is necessary to establish the diagnosis of cutaneous mucormycosis. The biopsy specimen should be taken from the center of the lesion and include subcutaneous fat, because molds frequently invade blood vessels of the dermis and subcutis, resulting in an ischemic cone at the skin surface [83]. Impression smears from the wound edges may also help with the diagnosis [69].

Culture is useful for identification of etiological agents and antifungal drug susceptibility testing. Mucorales are fast growing molds; thus, 24-h incubation on fungal media like Sabouraud agar and malt extract agar at 25–37 °C usually suffices for colony formation. A positive culture in a patient with no clinical symptoms should be interpreted with caution because of the possibility of environmental contamination. Besides the classic morphological identification of molds there are some metabolic markers that are important for species/genus identification, such as the ability to grow within certain temperature ranges and assimilation of different compounds as a sole carbon source.

Human pathogenic Mucorales differ with regard to carbon source utilization patterns [84], which could be used for species identification, either directly in culture-dependent assays or for the development of metabolite-based approaches. There are limited data on species differences in pathology/treatment owing to the very recent establishment of some of the aforementioned new taxa. Differential carbon source usage was reported for *Apophysomyces* with negative results for assimilation of D-galactose and other sugars [29] and positive for the members of six other genera of pathogenic Zygomycota, i.e., *Cunninghamella*, *Lichtheimia*, *Mucor*, *Rhizopus*, *Rhizomucor*, and *Syncephalastrum* [84]. Such differences might be used to develop identification tests.

Species identification can be confirmed by molecular methods based on the sequencing of a standard PCR product obtained with universal fungal ITS primers on DNA extracted from a culture, enabling reliable order/species level identification of the infecting fungus. In laboratories equipped with reference libraries techniques such as MALDI-TOF might also be possible from culture samples.

### Culture-independent methods: molecular diagnosis

Currently, there are no circulating antigen detection tests available. The promising 1,3 beta-D-glucan tests are commonly found to be negative in Mucorales. A double galactomannan and 1,3 beta-D-glucan test can be of help in excluding invasive aspergillosis or a double infection by *Aspergillus* and Mucorales. PCR-based tests of blood samples are not yet available. Consequently biological samples of the infected sites should be collected for histopathology and culture. Invasive diagnostic biopsy of infected tissue in sinus and lung infections should be performed. Blood and cerebrospinal fluid are usually culture-negative.

Culture-independent molecular techniques are being developed: some are complex and laborious [85] and others require sophisticated equipment and reference datasets. Zhao and colleagues describe an assay based on a combination of broad-range PCR amplification and reverse line blot hybridization (PCR/RLB) that enabled them to identify fungi from tissue samples in 7 h [85] with high specificity (no false-positives) and false-negatives solely for fungi not included in the reference. Additionally, their method works for concentrations of 1.8 × 10^−3^ ng/μl of genomic DNA.

More specific and sensitive molecular markers and secondary metabolite identification methods are still being developed.

MALDI-TOF diagnostics are currently based on reference datasets and require cultivation; however, a development of serum-based profiles is expected in the near future.

### Treatment

According to the largest meta-analysis performed by Roden and colleagues [8], lack of treatment had a fatal outcome, with only 3 % survival. A double treatment consisting of antifungal therapy combined with surgical intervention was most successful, leading to the survival of 70 % of patients.

Early diagnosis is the key factor for a successful outcome [8]. This is followed by surgical debridement and antifungal therapy. Necrotic tissue resulting from disease progression may require repeated excision as it prevents antifungal agent penetration [45]. Amphotericin B is the main drug used in the treatment of mucormycosis, entomophthoromycosis and mortierellomycosis. High therapeutic dosage should be achieved as soon as possible. Lipid formulations tend to be better tolerated, limiting the nephrotoxicity [86]. Currently, no other drug displays comparable activity against all three groups of fungi. The main antifungal drugs belong to five different groups: echinocandins, azoles, polyenes, allylamines, and pyrimidine analogs. The echinocandins (caspofungin, micafungin, and anidulafungin) inhibit the synthesis of 1,3-β-D-glucan, an essential component of the fungal cell wall. Azoles (fluconazole, hexaconazole, isavuconazole, itraconazole, posaconazole, ravuconazole, voriconazole) are lanosterol 14 alpha-demethylase inhibitors. Polyenes bind ergosterol, a cell membrane component (amphotericin B, nystatin, and natamycin). Allylamines inhibit squalene monooxygenase; among them only terbinafine is used against fungi. The main antifungal pyrimidine analog and thymidylate synthase inhibitor is flucytosine.

An evaluation of amphotericin B, ketoconazole, fluconazole, intraconazole, voriconazole, posaconazole, caspofungin, and flucytosine activity against most of the human pathogenic Mucorales: *Rhizopus* sp. *R. arrhizus*, *R. microsporus* var. *rhizopodiformis*, *R. microsporus* var. *micorsporus*, *Mucor* sp. *M. circinelloides* group, *Rhizomucor* sp., *Absidia* sp. *Absidia corymbifera*, and *Cunninghamella* sp. *Apophysomyces elegans* revealed that posaconazole is the second most promising drug [6]. A recent report from the 3rd European Conference on Infections in Leukemia (ECIL 3) provides a detailed guideline regarding the diagnosis and treatment and lists as yet unanswered questions [45].

#### Amphotericin B

Amphotericin B deoxycholate was introduced more than 50 years ago and become the gold standard of therapy for invasive mycoses [86, 87]. It has a broad spectrum activity against most fungal agents and serves as a reference in searches for novel antifungal drugs. The administration of AMB causes severe side effects, among them high nephrotoxicity, which leads to the development of less nephrotoxic lipid formulations. The latter can be administered in higher doses than the native drug. Comparative trials are performed between the more common liposomal amphotericin B (LAmB) and amphotericin B colloidal dispersion (ABCD), which is less frequently studied.

#### Posaconazole

Posaconazole is an exception among azoles, which display either moderate or no activity against most of the strains tested. Mucorales are in general susceptible to amphotericin B, but display variable response to posaconazole. A recent report from the SEIFEM and FUNGISCOPE [7, 88] registries evaluated the outcome of combined lipid formulation of amphotericin B (LAmpB) posaconazole (POS) therapy of invasive mucormycosis in 32 patients [88]. This evaluation was essential because of the limited number of cases in previous clinical reports and inconclusive previous studies that reported synergistic effects in vitro and no improvement relative to LAmpB monotherapy in mouse models. Pagano and colleagues conclude that a combined therapy should be considered in severe cases of invasive mucormycosis. Additionally, it is recommended to consider prophylaxis with posaconazole in at-risk groups. It is a broad spectrum drug that is protective against Zygomycota and *Fusarium*, *Penicillium*, *Histoplasma*, *Blastomyces*, *Coccidioides*, *Paracoccidioides*, *Sporotrix*, chromoblastomycosis, mycetoma, phaeohyphomycosis, including *Scedosporium apiospermum* and *Exophiala*, *Alternaria*, and *Bipolaris* spp. [89]. Among other things it has been used for the successful treatment of *Cunninghamella elegans* infection [90]. Furthermore, it is the most active drug in *A. elegans* treatment, has lower MIC than AmpB [91]. Saksenaeaceae (*Apophysomyces* and *Saksenaea*) seem to be most responsive to the antifungals posaconazole, itraconazole, and terbinafine [92], but not AmpB.

#### Voriconazole

Previous exposure of *Rhizopus oryzae* to voriconazole enhances virulence in animal models [93, 94]. It is discussed whether the co-occurrence of voriconazole application for antifungal prophylaxis in immunocompromised patients with the frequency of zygomycosis is of a causal nature [4]

#### Caspofungin

Reports of caspofungin treatment show discrepancies between in vitro and in vivo results. It is claimed that caspofungin facilitates the development of some zygomycoses in a similar manner to voriconazole and is associated with a worse outcome [65]. On the other hand, caspofungin shows synergy with AmpB in a mouse model [95]. There is a repeatedly confirmed paradox of caspofungin efficacy decreasing to control level when administered in higher doses in murine models [96].

#### Terbinafine

Recently, terbinafine has been successfully administered in patients with mucormycosis [83, 97, 98]. The drug has been primarily used against dermatophytes, but further usage included *Candida* and Zygomycota. Terbinafine was the most active compound (among amphotericin B, voriconazole, posaconazole, itraconazole, ravuconazole, terbinafine, and caspofungin) in an in vitro study of antifungal activity against five strains of *Cunninghamella bertholletiae* and four of *Cunninghamella echinulata* [99]. However, it is intriguing why in vivo studies did not include this compound. Nevertheless, the compound was successfully applied against *Basidiobolus ranarum* in a combination with itraconazole in a rhino-facial infection [100].

#### Combined antifungal therapy

Spellberg with colleagues [101] formulated three recommendations to introduce phase III clinical trials with combined therapy:

1. Improved survival in relevant animal models of mucormycosis
2. Available retrospective data that are concordant with the preclinical models both for efficacy and safety
3. Involvement of already approved therapeutic agents

The consortium proposed three potentially effective therapeutic combinations: lipid polyene and echinocandin, lipid polyene/posaconazole and deferasirox, and lipid polyene and posaconazole/isavuconazole. The aforementioned (L)AmpB and posaconazole combination was the most commonly used combined therapy in patients with hematological diseases reported from the SEIFEM and FUNGISCOPE registries. In the aforementioned studies LAmB+POS was administered when antifungal monotherapy failed to produce a response. These patients were treated with a monotherapy for a median time of 18 days before LAmB+POS combined therapy was introduced. A robust and consistent assessment of drug efficacy and the influence of combined therapy on patients’ outcome is limited by such factors as the limited number of cases, the lack of regular therapeutic drug monitoring, the uncontrolled impact of surgical debridement, and the burden of underlying diseases.

The concluding remark from the analyses of Pagano and colleagues [88] was that a combined antifungal treatment with LAmB+POS may be considered in patients with very aggressive forms of IM. This is the only drug combination that is consistently recommended by independent authorities. However, other combinations have been described as well.

In vitro studies as well as animal studies suggested a synergistic effect of deferasirox in combination with LAmB [102]. However, a randomized, double-blinded, placebo-controlled trial showed that patients with mucormycosis treated with deferasirox with LAmB had a higher mortality rate at 90 days than patients treated with LAmB with placebo. Authors emphasized that population imbalances in this small phase II study limit possible generalizations. Spellberg and co-authors concluded that although no obvious toxicities were identified, deferasirox cannot be recommended as part of an initial combination regimen for the treatment of mucormycosis [103].

From the theoretical point of view echinocandins should be active against Mucorales: *R. oryzae* produces the target enzyme for echinocandins [103]. In vitro studies show higher MICs for this group of drugs [104]. In a mouse model of disseminated mucormycosis a combination of caspofungin with ABLC [95] and LAmB with either micafungin or anidulafungin [105] markedly and synergistically improved survival compared with either monotherapy or placebo. Reed and colleagues conducted a retrospective study concluding that either ABLC or LAmB combined with CASP improved survival for patients with rhinocerebral mucormycosis compared with polyene monotherapy [106]. Micafungin was also successfully combined with LAmpB; however, systematic trials are lacking [107]. This effect was previously observed in an animal model of disseminated mucormycosis [105]. All these data “provide strong a rationale for a phase III, randomized double-blinded, placebo-controlled study to determine whether lipid polyene plus echinocandins are indeed superior to lipid polyene plus placebo therapy” [103].

### Alternative therapy

#### Iron chelators and hyperbaric oxygen

Both iron chelators (deferasirox and deferiprone) and hyperbaric oxygen have been used in some cases. However, they are not well documented enough to get positive recommendations from ECIL 3 [45].

#### Immunotherapy and immunostimulation

Adoptive immunotherapy with T cells after stimulation with cellular extracts of fungi may form the basis for future clinical trials. Simultaneous stimulation with *Aspergillus fumigatus*, *Candida albicans*, and *Rhizopus oryzae* extracts enables a generation of multi-specific T cells that recognize a wide variety of taxa [108]. These in vitro and in vivo results may represent a hope for allogeneic hematopoietic stem cell transplantation recipients with invasive fungal infection. However, there is no sufficient clinical evidence to formulate recommendations to use this approach in systemic zygomycosis [109].

The ECIL 3 suggests that the growth factors granulocyte colony-stimulating factor (G-CSF), granulocyte macrophage colony-stimulating factor (GM-CSF), and interferon-γ (IFN-γ) should be used in neutropenic patients with mucormycosis. Their administration in other patients needs to be studied further.

## Final remarks

The constantly growing number of patients susceptible to fungal infections emphasizes the critical need for better treatment and diagnostics. These can be developed based on rigorous meta-analyses of well-described case reports with proper taxon identification, treatment, and outcome description.

## Electronic supplementary material

Below is the link to the electronic supplementary material.

Supplementary Table S 1 (PDF 138 kb)
