# Supplementary material for: Biology, systematics, and clinical manifestations of Zygomycota infections
Source: Eur J Clin Microbiol Infect Dis. 2014 Mar 11;33(8):1273–87. doi: 10.1007/s10096-014-2076-0 (PMC4077243; doi:10.1007/s10096-014-2076-0)
Supplement: Supplementary file 1 — (PDF 138 kb) [file 10096_2014_2076_MOESM1_ESM.pdf]

Supplementary Table S1. Examples of case studies documenting Zygomycota infections.

**Taxon**	**Disease**
**Mucorales**	
Apophysomyces elegans	post-trauma [1], rhino-orbito-cerebral [2], renal [3]
Apophysomyces ossiformis	no reports
Apophysomyces variabilis	post-trauma [4]
Apophysomyces trapeziformis	post-trauma necrotizing [5][4]
Cokeromyces recurvatus	pneumonia [6], , diarrhea [7] [8]
Cunninghamella echinulata	sinus [9]
Cunninghamella elegans (syn. *Cunninghamella bertholletiae*)	pneumonia [10], systemic [11], cutaneous [12], rhino-orbital [13], endocardiovascular, and peritoneal [14]
Lichtheimia corymbifera (syn. *Absidia corymbifera, Mycocladus corymbifera*)	pneumonia [15], burns [16]
Mucor circinelloides	cutaneous [17][18], (with diabetes)
Mucor ellipsoideus	[19]
Mucor hiemalis	cutaneous [[20], [21]]
Mucor indicus	post-trauma GI-tract [22], systemic [23]
Mucor irregularis (syn. *Rhizomucor variabilis*)	rhinofacial [24, 25] , cutaneou s [26]
Mucor racemosus	
Mucor ramosissimus	cutaneous [27]
Mucor velutinosus	disseminated [28], [19]
Rhizomucor miehei	

Rhizomucor pusillus (syn. *Mucor pusillus*)	systemic [29]
Rhizopus arrhizus (syn. *Mucor rouxianus; R. oryzae; R. racemosus*)	
Rhizopus microsporus var. *azygosporus* (syn. *Rhizopus azygosporus*)	gastrointestinal [30], neonatal [31]
Rhizopus microsporus var. *microsporus*	
Rhizopus microsporus var. *oligosporus*	sinus ? [32]
Rhizopus microsporus var. *rhizopodiformis* (*Mucor rhizopodiformis; Rhizopus rhizopodiformis*)	disseminated [32], liver [33]
Rhizopus schipperae	
Rhizopus stolnifer	
Saksenaea vasiformis	post-trauma
Saksenaea erythrospora	post-trauma [34]
Syncephalastrum racemosum (syn. *Syncephalastrum cinereum, Syncephalastrum elegans, Syncephalastrum fuliginosum, Syncephalastrum javanicum,* *Syncephalastrum nigricans*)	Cutaneous [35]
**Entomophtorales**	
Basidiobolus ranarum (syn. *Basidiobolus meristosporus, Basidiobolus haptosporus, Basidiobolus heterosporus*)	gastrointestinal [36] cuteaneous [37], subcutaneous [38], disseminated [39]
Conidiobolus coronatus (syn. *Boudierella*	Rhinofacial subcutaneous [40],

coronata, Conidiobolus villosus, Delacroixia coronata, Entomophthora coronate)	
Conidiobolus lamprauges	Disseminated [41]
**Mortierellales**	
Mortierella wolfii	
Mortierella polycephala	pulmonary infections in cattle [42]

Supplementary references:

1. Andresen D, Donaldson A, Choo L, Knox A, Klaassen M, Ursic C, Vonthethoff L, Krilis S, Konecny P (2005) Multifocal cutaneous mucormycosis complicating polymicrobial wound infections in a tsunami survivor from Sri Lanka. Lancet 365:876–878.

2. Liang KP, Tleyjeh IM, Wilson WR, Roberts GD TZ (2006) Rhino-orbitocerebral mucormycosis caused by *Apophysomyces elegans.* J Clin Microbiol 44:892–8.

3. Marak RSK, Misra R, Ansari MS, Dixit A, Poornima, Prasad KN, Dhole TN (2010) Successful medical management of renal zygomycosis: a summary of two cases and a review of the Indian literature. Med Mycol 48:1088–95.

4. Dela Cruz WP, Calvano TP, Griffith ME, White CE, Kim SH, Sutton DA, Thompson EH, Fu J, Wickes BL, Guarro J, Hospenthal DR (2012) Invasive *Apophysomyces variabilis* infection in a burn patient. J Clin Microbiol 50:2814–7.

5. Etienne KA, Gillece J, Hilsabeck R, Schupp JM, Colman R, Lockhart SR, Gade L, Thompson EH, Sutton DA, Neblett-Fanfair R, Park BJ, Turabelidze G, Keim P, Brandt ME, Deak E, Engelthaler DM (2012) Whole genome sequence typing to investigate the *Apophysomyces* outbreak following a tornado in Joplin, Missouri, 2011. PLoS One 7:e49989.

6. Ryan LJ, Ferrieri P, Powell RD, Paddock CD, Zaki SR, Pambuccian SE (2011) Fatal *Cokeromyces recurvatus* pneumonia: report of a case highlighting the potential for histopathologic misdiagnosis as coccidioides. Int J Surg Pathol 19:373–6.

7. Tsai TW, Hammond LA, Rinaldi M, Martin K, Tio F, Maples J, Freytes CO, Roodman GD (1997) C*okeromyces recurvatus* infection in a bone marrow transplant recipient. Bone Marrow Transplant 19:301–2.

8. Alvarez OA, Maples JA, Tio FO, Lee M (1995) Severe diarrhea due to *Cokeromyces recurvatus* in a bone marrow transplant recipient. Am J Gastroenterol 90:1350–1.

9. Leblanc RE, Meriden Z, Sutton DA, Thompson EH, Neofytos D, Zhang SX (2013) *Cunninghamella echinulata* causing fatally invasive fungal sinusitis. Diagn Microbiol Infect Dis 76:506–509.

10. Gupta R, Goel N, Gupta A, Gupta KB, Chaudhary U, Sood S (2011) A rare fungal infiltration of lungs in a healthy young girl. Case Rep Pulmonol 2011:917089.

11. Hsieh T-T, Tseng H-K, Sun P-L, Wu Y-H, Chen G-S (2013) Disseminated zygomycosis caused by *Cunninghamella bertholletiae* in patient with hematological malignancy and review of published case reports. Mycopathologia 175:99–106.

12. Motohashi K, Ito S, Hagihara M, Maruta A, Ishigatsubo Y, Kanamori H (2009) Cutaneous zygomycosis caused by *Cunninghamella bertholletiae* in a patient with chronic myelogenous leukemia in blast crisis. Am J Hematol 84:447–8.

13. Righi E, Giacomazzi CG, Lindstrom V, Albarello A, Soro O, Miglino M, Perotti M, Varnier OE, Gobbi M, Viscoli C, Bassetti M (2008) A case of *Cunninghamella bertholettiae* rhino-cerebral infection in a leukaemic patient and review of recent published studies. Mycopathologia 165:407–10.

14. Gomes MZR, Lewis RE, Kontoyiannis DP (2011) Mucormycosis caused by unusual mucormycetes, non-Rhizopus, -Mucor, and -Lichtheimia species. Clin Microbiol Rev 24:411–45.

15. Kleinotiene G, Posiunas G, Raistenskis J, Zurauskas E, Stankeviciene S, Daugelaviciene V, Machaczka M (2013) Liposomal amphotericin B and surgery as successful therapy for pulmonary *Lichtheimia corymbifera* zygomycosis in a pediatric patient with acute promyelocytic leukemia on antifungal prophylaxis with posaconazole. Med Oncol 30:433.

16. Constantinides J, Misra A, Nassab R, Wilson Y (2008) *Absidia corymbifera* fungal infection in burns: a case report and review of the literature. J Burn Care Res 29:416–9.

17. Dizbay M, Adisen E, Kustimur S, Sari N, Cengiz B, Yalcin B, Kalkanci A, Gonul II, Sugita T (2009) Fungemia and cutaneous zygomycosis due to *Mucor circinelloides* in an intensive care unit patient: case report and review of literature. Jpn J Infect Dis 62:146–8.

18. Iwen PC, Sigler L, Noel RK, Freifeld AG (2007) *Mucor circinelloides* was identified by molecular methods as a cause of primary cutaneous zygomycosis. J Clin Microbiol 45:636–40.

19. Alvarez E, Cano J, Stchigel AM, Sutton DA, Fothergill AW, Salas V, Rinaldi MG, Guarro J (2011) Two new species of *Mucor* from clinical samples. Med Mycol 49:62–72.

20. Prevoo RL, Starink TM, de Haan P (1991) Primary cutaneous mucormycosis in a healthy young girl. Report of a case caused by *Mucor hiemalis* Wehmer. J Am Acad Dermatol 24(5 Pt 2):882–5.

21. Costa AR, Porto E, Tayah M, Valente NY, Lacaz C da S, Maranhão WM, Rodrigues MC (1990) Subcutaneous mucormycosis caused by *Mucor hiemalis* Wehmer f. luteus (Linnemann) Schipper 1973. Mycoses 33:241–6.

22. Deja M, Wolf S, Weber-Carstens S, Lehmann T-N, Adler A, Ruhnke M, Tintelnot K (2006) Gastrointestinal zygomycosis caused by *Mucor indicus* in a patient with acute traumatic brain injury. Med Mycol 44:683–7.

23. Ter Borg F, Kuijper EJ, van der Lelie H (1990) Fatal mucormycosis presenting as an appendiceal mass with metastatic spread to the liver during chemotherapy-induced granulocytopenia. Scand J Infect Dis 22:499–501.

24. Hemashettar BM, Patil RN, O’Donnell K, Chaturvedi V, Ren P, Padhye AA (2011) Chronic rhinofacial mucormycosis caused by *Mucor irregularis* (*Rhizomucor variabilis*) in India. J Clin Microbiol 49:2372–5.

25. Li DM, Lun L De (2012) *Mucor irregularis* infection and lethal midline granuloma: a case report and review of published literature. Mycopathologia 174:429–39.

26. Lu X, Liu Z, Shen Y, She X, Lu G, Zhan P, Fu M, Zhang X, Ge Y, Liu W (2009) Primary cutaneous zygomycosis caused by *Rhizomucor variabilis*: a new endemic zygomycosis? A case report and review of 6 cases reported from China. Clin Infect Dis 49:e39–43.

27. Weitzman I, Della-Latta P, Housey G, Rebatta G (1993) *Mucor ramosissimus* Samutsevitsch isolated from a thigh lesion. J Clin Microbiol 31:2523–5.

28. Sugui JA, Christensen JA, Bennett JE, Zelazny AM, Kwon-Chung KJ (2011) Hematogenously disseminated skin disease caused by *Mucor velutinosus* in a patient with acute myeloid leukemia. J Clin Microbiol 49:2728–32.

29. Hadaschik E, Koschny R, Willinger B, Hallscheidt P, Enk A, Hartschuh W (2012) Pulmonary, rhino-orbital and cutaneous mucormycosis caused by *Rhizomucor pusillus* in an immunocompromised patient. Clin Exp Dermatol 37:355–7.

30. Roussy J-F, Allard C, St-Germain G, Pépin J (2012) Gastrointestinal Mucormycosis following a Streptococcus pyogenes Toxic Shock Syndrome in a Previously Healthy Patient: A Case Report. Case Rep Infect Dis 2012:476719.

31. Schipper MA, Maslen MM, Hogg GG, Chow CW, Samson RA (1996) Human infection by *Rhizopus azygosporus* and the occurrence of azygospores in Zygomycetes. J Med Vet Mycol 34:199–203.

32. Ribeiro LC, Wanke B, da Silva M, Dias LB, Mello R, Canavarros FAPB, Leite-Jr DP, Hahn RC (2012) Mucormycosis in Mato Grosso, Brazil: a case reports, caused by *Rhizopus microsporus* var. *oligosporus* and *Rhizopus microsporus* var. *rhizopodiformis*. Mycopathologia 173:187–92.

33. Sedlacek P, Vavra V, Masova I, Codl D, Laznickova T, Malaskova L, Nyc O, Stary J (2009) Successful therapy with ABLC, surgery and posaconazole for *Rhizopus microsporus* var. *rhizopodiformis* liver eumycetoma in a child with acute leukaemia. Mycoses 52:276–9.

34. Hospenthal DR, Chung KK, Lairet K, Thompson EH, Guarro J, Renz EM, Sutton DA (2011) *Saksenaea erythrospora* infection following combat trauma. J Clin Microbiol 49:3707–9.

35. Pavlović MD, Bulajić N (2006) Great toenail onychomycosis caused by *Syncephalastrum racemosum*. Dermatol Online J 12:7.

36. Geramizadeh B, Foroughi R, Keshtkar-Jahromi M, Malek-Hosseini S-A, Alborzi A (2012) Gastrointestinal basidiobolomycosis, an emerging infection in the immunocompetent host: a report of 14 patients. J Med Microbiol 61(Pt 12):1770–4.

37. Mendiratta V, Karmakar S, Jain A, Jabeen M (2012) Severe cutaneous zygomycosis due to *Basidiobolus ranarum* in a young infant. Pediatr Dermatol 29:121–3.

38. Ramesh V, Ramam M, Capoor MR, Sugandhan S, Dhawan J, Khanna G (2010) Subcutaneous zygomycosis: report of 10 cases from two institutions in North India. J Eur Acad Dermatol Venereol 24:1220–5.

39. Van den Berk GEL, Noorduyn LA, van Ketel RJ, van Leeuwen J, Bemelman WA, Prins JM (2006) A fatal pseudo-tumour: disseminated basidiobolomycosis. BMC Infect Dis 6:140.

40. Isa-Isa R, Arenas R, Fernández RF, Isa M (2012) Rhinofacial conidiobolomycosis (entomophthoramycosis). Clin Dermatol 30:409–412.

41. Kimura M, Yaguchi T, Sutton DA, Fothergill AW, Thompson EH, Wickes BL (2011) Disseminated human conidiobolomycosis due to *Conidiobolus lamprauges*. J Clin Microbiol 49:752–6.

42. Scholz HD, Meyer L (1965) *Mortierella polycephala* as a causative agent of pulmonary mycosis in cattle. Berl Munch Tierarztl Wochenschr 78:27–30.
